# Barriers and facilitators to preventive interventions for the development of obstetric fistulas among women in sub-Saharan Africa: a systematic review

**DOI:** 10.1186/s12884-018-1787-0

**Published:** 2018-05-10

**Authors:** Eniya Lufumpa, Lucy Doos, Antje Lindenmeyer

**Affiliations:** 1 Institute of Applied Health Research, University of Birmingham, Edgbaston, Birmingham B15 2TT UK; 2 Institute for Research into Superdiversity, University of Birmingham, Edgbaston, Birmingham B15 2TT UK

**Keywords:** Obstetric fistula, Preventive interventions, Barriers, Facilitators, Basic and comprehensive emergency obstetric care

## Abstract

**Background:**

Obstetric fistula is a debilitating childbearing injury that results from poorly managed obstructed labour, leading to the development of holes between the vagina and bladder and/or rectum. Effects of this injury are long-lasting, as women become incontinent and are often marginalised from their communities. Despite continuous occurrence of this injury in lower-income countries, it is preventable, as evidenced in high-income countries. This systematic review aims to identify and understand barriers and facilitators to interventions aimed at the prevention of obstetric fistulas in sub-Saharan African women.

**Methods:**

Electronic databases and grey literature were searched. We included studies written in English that discussed interventions to prevent obstetric fistulas implemented in sub-Saharan Africa, and their associated barriers and facilitators. Quality of the studies was assessed, and data including: country of implementation, preventive interventions, and barriers and facilitators to the interventions were extracted. They were then categorised based on the *Three Phase Delay Model*.

**Results:**

Our search yielded 537 studies, of which 18 were included from sub-Saharan countries including Ethiopia, Nigeria, and Zambia. The most noted barrier to prevention addressed the first phase of delay: the decision to seek care, particularly lack of awareness of the dangers of unsupervised labours. The most noted facilitator addressed the decision to seek care and the quality of care received at a facility, through partnerships between health facilities and governments, and other organisations that provided both financial and resource support.

**Conclusion:**

Despite being categorised by the three phases of the delay model, barriers and facilitators were found to play a role in multiple phases. The topic of obstetric fistula needs to be researched more extensively, particularly the effectiveness of preventive interventions.

**Electronic supplementary material:**

The online version of this article (10.1186/s12884-018-1787-0) contains supplementary material, which is available to authorized users.

## Background

Obstetric fistula is a debilitating childbearing injury seen only in lower-income countries. It results predominantly from obstructed labour, but can also be iatrogenic and result from errors during surgeries such as caesarean sections. It represents a major public health issue for women and their communities, particularly in sub-Saharan Africa and Southeast Asia [1]. This injury results in holes between the vagina and bladder (vesicovaginal fistula), and/ or vagina and rectum (rectovaginal fistula) [2]. These holes lead to incontinence of urine, and/ or faeces [3]. These have secondary effects such as the marginalisation and social exclusion of women from within their communities [4]. Social exclusion also decreases their likelihood of seeking treatment because they are less likely to be made aware of treatment options available to them [5]. Not seeking treatment results in difficulties in estimating the number of women currently suffering from this injury due to under-diagnosis. Current data reflect fistulas that have been accounted for mainly through clinical records, and there are still thousands of women who have yet to be accounted for [6, 7].

With an estimated number of more than two million women living with untreated fistulas worldwide, every year between 50,000 and 100,000 new cases of obstetric fistula occur [4]. Obstetric fistulas can be treated with reconstructive intravaginal surgery; however, the majority of affected women are unable to afford this treatment [8]. Conversely, fistulas are easily prevented through timely access to competent emergency obstetric care (EmOC) at the onset of labour complications such as prolonged labour [9]. As evidenced in high-income countries, this injury is completely preventable if proper measures are taken to educate women and healthcare providers about identifying and managing obstetric complications, while concurrently strengthening existing health systems within affected countries so as to ensure provision of adequate maternal care for all pregnant women [10]. This study focuses on prevention as the main means of ultimately eradicating this injury.

While preventive interventions in sub-Saharan Africa have already been identified in a previous systematic review by Banke-Thomas et al., included in this study, there is a need for a thorough assessment of the barriers to and facilitators of these interventions [11]. This is a critical component in making preventive interventions more accessible and effective. It is this knowledge gap which this systematic review aims to address.

## Methods

**Fig. 1:**
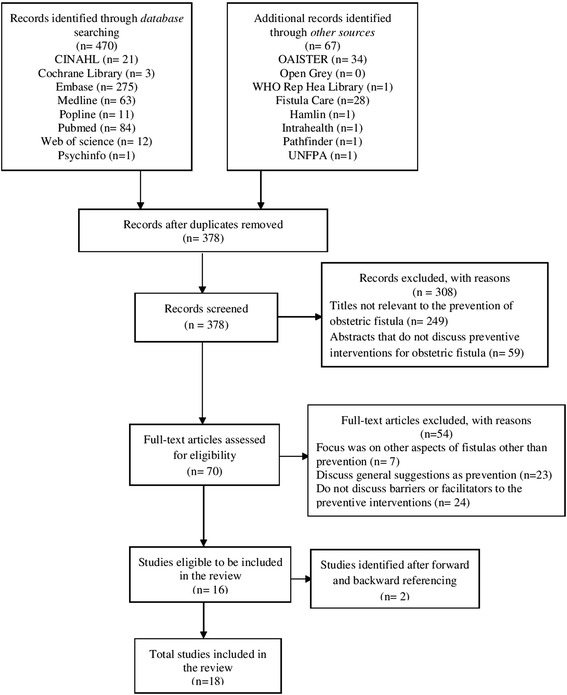
PRISMA flow diagram of study inclusion process

Using the following databases: PubMed, EMBASE, MEDLINE, Cochrane Library, CINAHL, POPLINE, PsycINFO, and Web of Science for articles that identified barriers to and facilitators of interventions that aim to prevent the development of obstetric fistulas in sub-Saharan African women. Additional resources included official documents and grey literature—which included databases and websites of relevant organisations such as EngenderHealth, FistulaCare and the World Health Organization (WHO) (see Fig. 1). Search terms included both MESH terms and free text including: ‘obstetric fistula’, ‘prevention’, and ‘sub-Saharan Africa’. A variation of these terms was used in the various databases listed above (Additional file 1).

We included articles written in English that met the following criteria: i) the setting of sub-Saharan Africa; ii) an intervention with the aim of preventing fistulas or its leading cause— obstructed labour— as well as its barriers and facilitators; and iii) fistulas that are a result of childbirth. The focus on sub-Saharan Africa is due to the highest prevalence in that continent; furthermore we also focused on obstetric fistulas as these are the most common types of fistulas within this region and allowed us to examine this topic with a context-specific approach [1]. We excluded studies that: i) were published in a language other than English; ii) discussed fistulas other than obstetric fistulas—vesicovaginal and rectovaginal—such as urethral fistulas, enterovaginal fistulas, and cervical fistulas; iii) discussed fistulas that are due to causes other than obstructed labour, for example, sexual trauma or radiation; and iv) focused on aspects other than prevention such as treatment, repair outcomes, experiences of living with this injury.

Articles were selected by two reviewers (EL, LD) using the process proposed by PRISMA, with a third reviewer resolving discrepancies (AL). We appraised included studies before the synthesis of the data, using either the relevant CASP checklist for qualitative studies or the AMSTAR checklist for systematic reviews. Descriptive studies were not appraised, as there was no applicable appraisal tool.

Reporting of the results was framed by the *Three Phase Delay Model*, proposed by Thaddeus and Maine [12]. The model identifies obstacles to the provision and timely utilisation of obstetric care—decision to seek care (first phase delay), accessibility of healthcare facilities (second phase delay), and receiving adequate care (third phase delay)— which are also applicable to preventive strategies.

## Results

The search yielded a total of 537 studies, of which 70 studies were fully retrieved and read. A total of 16 studies met our inclusion criteria. These studies were then forward and backward referenced, which further identified two relevant studies, resulting in 18 studies identified as meeting the inclusion criteria (see Fig. 1).

### Characteristics of included studies

All 18 included studies reflected: sub-Saharan Africa as a region (6), Nigeria (4), West Africa (1), Ethiopia (1), Eritrea (1), Guinea (1), Niger (1), Sierra Leone (1), Uganda (1) and Zambia (1), respectively. Nine studies were qualitative, eight descriptive, and one was a systematic review. Both barriers and facilitators were discussed to some extent in all 18 studies, with the exception of the study by Markos and Bogale which focused solely on barriers [13]. Barriers and facilitators were then characterised according to the Three Phase Delay Model. It is important to note that some of the included preventive interventions, mentioned in Table 1, do not have the primary aim of preventing fistulas but are instead basic and comprehensive maternity care that are not always readily available or accessible in lower-income countries, and as such were included as preventive interventions by the authors [11].

**Table 1:** Characteristics of included studies reporting on implemented fistula prevention interventions

Author(s)	Year	Country/ region of study	Preventive intervention implemented	Phase applicable to intervention
Bacon	2003	sub-Saharan Africa	Improving access to adequate medical care; Health education programs	All three phases
Banke-Thomas et al.	2014	sub-Saharan Africa	Community and Facility based interventions	All three phases
Fistula care^a^	2010	Guinea (Kissidougou)	Village safe motherhood committees; Financial partnerships; Maternal waiting homes; and Market Towns and Local Resource Mobilisation project	All three phases
Fistula care (a)^a^	2011	Sierra Leone	Aberdeen’s Women’s Centre: counselling, family planning, and a maternity care unit for pregnancy care, labour and delivery, and postpartum recovery (services to prevent fistulas)	All three phases
Fistula care (b)^a^	2011	sub-Saharan Africa	Partograph	Phase 3: Receiving adequate care
Fistula care^a^	2013	Uganda	Partograph	Phase 3: Receiving adequate care
Gerten et al.	2009	Nigeria	Patient educational brochure at a vesicovaginal hospital	Phase 1: Decision to seek care
Levin and Kabagema	2011	sub-Saharan Africa	Partograph	Phase 3: Receiving adequate care
Markos and Bogale	2015	Ethiopia (Bale zone)	Partograph	Phase 3: Receiving adequate care
Miller et al.	2005	sub-Saharan Africa	Fistula prevention centres; Community-based preventions; Maternal waiting homes; and Training course about screening for risk of fistula	All three phases
Nathan et al.	2009	West Africa	Femme pour Femme, community healthcare insurance plan	All three phases
Ngoma	2011	Zambia	EmOC and Safe Motherhood Actions Groups (SMAGs); and Income generating activities (IGA)	All three phases
Ojanuga	1991	Nigeria	Community health education programs; organizing transport for pregnant women in need; and training traditional birth attendants	All three phases
Ojanuga	1992	Nigeria	Health education programs	Phase 1: Decision to seek care
Seim et al.	2014	Niger	Community-mobilization program that arrange transport for women who experience complicated labours	All three phases
Tahzib	1989	Nigeria	Safe motherhood initiatives not specified but aimed at encouraging hospital deliveries, and the improvement of the perception of facilities by women seeking help	All three phases
Turan et al.	2007	Eritrea	Transport of women to healthcare facilities	Phase 2: Accessibility of care
UNFPA	2013	sub-Saharan Africa	Global midwifery program; All-terrain motorbikes (Women and Health Alliance International)	Phase 2: Accessibility of care; Phase 3: Receiving adequate care

^a^A six-year fistula repair and prevention program managed and implemented by Engender Health from 2007 to 2013

### Barriers

#### Phase one: Decision to seek care

Barriers were discussed in all studies (Table 2). The most frequently noted barrier was lack of awareness, as cited in four studies [14–17]. One study found that communities where fistulas are present were found to have limited knowledge of health issues, even more so with regard to the dangers of unsupervised births [16]. When a pregnant woman or members of her support system within her community were not aware of programmes that promote safe motherhood practices, they would not use the services that have been made available to them [16]. Two studies from Nigeria pointed out that this was rooted in the fact that fistulas are most prevalent in remote areas where there are high levels of illiteracy which are exacerbated by low levels of education [14, 16]. As revealed through the use of an educational brochure in one study, Gerten et al. discuss the limited effectiveness of educational brochures published in English as some participants lived in remote areas and had very limited education resulting in their ability to only read and communicate in their native language, rendering brochures ineffective [14].

**Table 2:** Barriers to interventions aimed at the prevention of obstetric fistulas categorised by the three phases of delay

Phase one: *Decision* to seek care	Phase two: *Reaching* a facility or preventive intervention	Phase three: *Receiving* adequate care through a preventive intervention
• Lack of awareness about health and preventive interventions ○ Ignorance among the villagers of the dangers associated with unsupervised delivery for women who are at risk ○ Negative experiences of other women at healthcare facilities ○ Illiteracy • Lack of access to preventive interventions • Lack of financial resources serve as a major disincentive to the use of modern health facilities • Reluctance of women to be away from their homes for an undetermined period of time • Language barrier, dependence on translation of a brochure into the reader’s native language	• Preventive strategies regarding birth plans are lagging • Lack of infrastructures such as paved roads, piped water, and electricity. ○ Worsens accessibility during he rainy and harvest seasons • Lack of transport ○ Large distances from the villages to healthcare facilities • Lack of financial resources to pay for transport • Lack of ambulance services and portable oxygen • Limited referral systems i.e. when emergency transport isn’t available	• Perception, healthcare practitioners view women with fistulas as a ‘nuisance’ and ‘embarrassment’ ○ Affects their attitude towards them and in turn the experience of the patient • Limited services and manpower ○ Doctors are preoccupied with high-tech practices, leaving their units overwhelmed with obstetric emergencies ○ Overworked staff ○ Staff shortages and high attrition rates • Lack of skilled healthcare providers ○ High staff turnover at maternity units which results in the loss of valuable skills and training investments ○ Absence of supervisory staff • Lack of financial resources, which leaves the facilities rarely self-sufficient • Lack of reimbursement for village practitioners • Improper/ limited use of the partograph ○ Lack of essential supplies and equipment needed ○ Lack of training

Lack of awareness was not limited to the knowledge of preventive interventions, but also applied to the perception of services provided at healthcare facilities [17]. Even when women were aware of preventive interventions some were still reluctant to seek care for obstetric complications due to their views regarding healthcare facilities which were shaped directly by the experiences of other rural women who sought care at a facility [17]. The author further pointed out that this served as a barrier in cases when a woman had a negative experience at a healthcare facility.

#### Phase two: Accessibility of care

Accessibility of preventive interventions was identified as a barrier in seven studies [14, 15, 17–21]. In Ojanuga’s study, longer distances between remote communities— where fistulas are prevalent—and preventive services—usually in urban settings—in Nigeria were identified as a phase two delay [16]. A further two studies based on Niger and Nigeria concluded that in areas where fistulas are most prevalent, dilapidated infrastructure made traveling to reach care nearly impossible [14, 20]. One study noted that conditions such as these were exacerbated during the rainy and harvest seasons, which further limited mobility and transport opportunities [18]. Moreover, Ojanuga’s study highlighted that traveling such great distances and rough terrains were extremely costly and difficult to arrange, due to their limited financial resources [15].

As highlighted in five studies, financial limitations served as a barrier to women accessing preventive interventions [14–16, 22, 23]. Nathan et al. pointed out that within West Africa, limited financial resources was the most frequently noted barrier to intrapartum care, citing the example of Benin, where the yearly income per capita is about $1100 USD, which makes transport and medical care costs unaffordable [23].

#### Phase three: Receiving adequate care

Receiving care through preventive facilities and available services was the most frequently discussed issue as is evidenced in seven studies [11, 13, 17, 22, 24–26]. As discussed in five studies, limited manpower, and shortage of skilled healthcare workers was an important factor in third phase delay [11, 20, 24–26]. Two studies highlighted that this was further heightened by the lack of basic and essential supplies and equipment including access to running water and electricity, which had proven to be unreliable [17, 20, 24]. This was often the case as they had financial limitations. Two studies showed how this directly affected a facility as their financial resources were meant to maintain services through the provision of basic equipment and supplies, as well as the training and payment of the service providers [19, 22]. Fistula Care, and Seim et al. reported that this included transport systems that are meant to be provided through preventive interventions (Table 2) [19, 20].

Four studies highlighted the importance of a partograph—a pre-printed recording sheet on which progress of labour is documented and monitored by a healthcare provider— and its ability to greatly reduce maternal and foetal morbidity and mortality, when properly used [13, 19, 24, 25]. The most commonly cited barrier to the use of a partograph was the improper and poor use of it due to the difficulty of its use [13, 19, 24, 25]. One study highlighted that this was due to differences in the versions of partographs, as well as incompetent monitoring and recording of the necessary indicators [24]. Moreover, minimal training on how to use a partograph was referenced in the study [24]. This barrier was compounded with the lack of essential supplies such as the partograph forms, clocks, blood pressure monitors etc. further rendering this preventive intervention impossible to use effectively [24].

### Facilitators

#### Phase one: Decision to seek care

Facilitators were discussed in 17 studies (Table 3). Increased awareness of maternal and child morbidities and how to prevent them, was noted as a facilitator in seven studies, particularly through health education programmes [14–16, 20–22, 27]. As Gerten et al. and Turan et al. reported, conducting programmes in the local languages addressed potential language barriers [14, 21]. The study conducted by Turan et al. also highlighted the use of figures and pictures as an aid which directly addressed the issue of illiteracy, which was often common in the areas where fistulas occur [21].

**Table 3:** Facilitators to interventions aimed at the prevention of obstetric fistulas, categorised by the three phases of delay

Phase one: *Decision* to seek care	Phase two: *Reaching* a facility or preventive intervention	Phase three: *Receiving* adequate care through a preventive intervention
• Women with successful treatments acting as ambassadors and advocates for healthcare facilities ○ Women are provided with training on public speaking and interpersonal communication skills • Increased awareness within communities through training ○ About maternal/ child morbidities, and the importance of seeking care • Community involvement ○ Volunteer coordinator provides SMAGs with technical support, and schedule activities and training ○ Increased involvement of men, community leaders, and religious leaders, as they are decision-makers within these communities • Financial support ○ Allowing women to participate in income-generating activities ○ Free healthcare services for pregnant women and children under 5 years old	• Financial support ○ Reimbursement for transport ○ Insurance plan that provides transport costs ○ Community generating money to assist with transport costs • Assistance with transport ○ A politician procured an ambulance, which facilitated the evacuation of labouring women in need ○ All-terrain motorbikes to facilitate transport to healthcare facilities • Volunteers initiate evacuation by phoning a midwife at a facility that has an ambulance available • Volunteers arrange transport to the closest facility • Relocation of midwives in rural areas where the most at-risk women and girls reside • Improved mobile coverage to arrange evacuation	• Mobilisation of recognised experts • Training ○ On the local needs as a means of improving the morale for the provision of preventive care ○ On the improved use of the partograph ○ Of patients so they educate women and their local communities when they return home • Financial support from international foundations and organisations ○ Insurance plan that covers medical costs for pregnant women • Partnerships that provide funds for research, the purchase of essential equipment, and the development of basic infrastructure ○ Donation of supplies and volunteers’ time, which improves adequate staffing, space, equipment, and essential medication • Employment of more midwives • Mobile prenatal clinics serve remote villages

Another key element noted in eight studies based on Eritrea, Guinea, Niger, Nigeria, and sub-Saharan Africa as a region, was the involvement of key members within communities [11, 15–18, 20–22]. This included the involvement of men, and religious and community leaders, as they are the decision-makers and gatekeepers of their societies [16, 20, 22]. Miller et al. highlighted the Tostan program in which traditional and village leaders come together to publicly advocate the rights of girls and women, as well as the end of harmful cultural practices [22]. Also noted in five studies was the involvement of women who had previously used the service [11, 15, 17, 18, 21]. Women who had successful treatments would serve as advocates and community educators to encourage women to attend healthcare facilities when they experience complications during labour.

#### Phase two: Accessibility of care

Facilitating the accessibility of preventive interventions was addressed in seven studies [15, 18, 20, 22, 23, 26, 27]. Four studies discussed efforts by healthcare facilities and individuals to improve access to health services [15, 20, 22, 28]. UNFPA highlighted the use of motorbike ambulances within countries from West to East Africa, which are low cost and quickly transport women in labour to healthcare facilities [28]. In cases where emergency transport services were not available, three studies mentioned the use of mobile prenatal clinics and maternity waiting homes [15, 18, 22]. The use of mobile clinics addressed delays in receiving care, while maternity waiting homes provided pregnant women who have high-risk pregnancies with a place to stay that is within the vicinity of a facility that offers EmOC, if necessary [18, 22].

Financial support also served as a facilitator to the accessibility of care, as noted in five studies [15, 18, 20, 23, 27]. The studies discussed community-led programs—in the West African region, Guinea, Nigeria, and Zambia, respectively—that acted as insurance plans and provided financial support for women in need. Seim et al. identified a program in Niger that subsidised the cost of fuel that ambulances used to transport pregnant women to hopsitals [20]. Two studies carried out by Fistula Care and Ngoma highlighted the importance of Safe Motherhood Committees [18, 27]. These committees participate in income-generating activities which provide financial support for women needing emergency care. Fistula Care pointed out the importance of income-generating activities in the Guinean community of Kissidougou through which a minimum of 5% of generated income is allocated to their local fistula programs [18].

#### Phase three: Receiving adequate care

Receiving adequate care was facilitated through financial support from partnerships with the government, and international organisations and foundations, as mentioned in three studies [17, 19, 25]. Financial support was provided to hospitals and other preventive interventions, particularly with essential equipment needed to provide care for women with labour complications.

Miller et al. and Seim et al. note the employment of more midwives, and their relocation to rural communities where fistulas are most prevalent as a facilitator [20, 22]. Moreover, two studies discussed the use of mentoring and training programs as a means of improving the competence of care providers as well as their morale [17, 25]. Mentoring and training was also provided on the use of the partograph—a pre-printed recording sheet on which the progression of labour is documented and monitored by a healthcare provider. This training would enable healthcare providers to take timely and appropriate decisions in response to the progression of labour [25].

## Discussion

Numerous barriers and facilitators were identified, and categorised by the *Three Phase Delay Model*– the decision to seek preventive care, accessing preventive care, and receiving adequate preventive care. Our analyses demonstrate that despite being categorised by the three phases of the delay model, barriers and facilitators were found not to be limited to one phase. More specifically, barriers— limited finances, limited education and awareness, and location of services— were also found to be interlinked. Fistulas are most prevalent in remote communities far from educational and health facilities. Characteristic of these areas are low levels of education and financial instability, as these communities rely on subsistence farming [16]. We also found that facilitators discussed in the literature, unlike barriers, were not found to be as clearly interlinked as they were more diverse. The main facilitators to interventions aimed at the prevention of fistulas— increased awareness of maternal and child morbidities, involvement of key members of the community, and financial support—were also not limited to one phase, but instead affected all three phases of delay. Accordingly, they were examined across phases.

### Barriers

Despite numerous countries adopting recommendations of United Nations agencies and the African Union to subsidize the cost of maternal care, women are still required to pay for some costs [29]. Our review highlighted limited finances as the most cited barrier, which was echoed in relevant studies as affecting all three phases of delay [29]. Our findings mirror the study of Melberg et al. in rural Burkina Faso, and found that expectant women were reluctant to leave their homes for an unknown duration due to their inability to attend to their responsibilities at home and participate in income-generating activities [30]. Additionally, limited finances led to a lack of basic necessities such as scissors, blood pressure monitors, and ambulances which greatly impaired the services that health facilities could provide. They further point out that these are exacerbated by a lack of basic amenities such as beds, adequate lighting, or running water which makes the provision of quality care difficult as issues such as sterility arise [30].

Our systematic review confirms that limited education was found not only with regard to people living in remote communities where fistulas are found, but also applied to the skills of health care providers. Our findings concur with Wall’s study of fistula patients in Jos, Nigeria, which reported that they were unaware of obstetric care available to them at the time [31]. Our study is also in line with Melberg et al. who attributed lack of skilled health care workers to high turnover rates within maternity units, which leads to the loss of skills and training investments [30]. Their study also reinforced barriers as being interlinked, as they pointed out that childbearing injuries are most common in women who are living far away from health centres and consequently may be unable to understand advantages of care at health facilities [30]. Another barrier linked to distance was the inability to afford transport from the areas they reside to health care facilities. Lori et al. point out that it is often worsened by the location of health facilities, being situated in more populated and urban areas [32]. Due to these longer distances, transport costs are unaffordable and this is compounded by unreliable transport.

Most barriers to receiving care were noted with regard to the partograph, specifically its improper use. Improper use was noted as a result of different versions of the partograph used in various facilities as well as inadequate monitoring of indicators required to use a partograph. Health care providers were unable to constantly monitor progress of labour [30]. This also resulted from lack of essential supplies required to monitor the necessary indicators, such as partograph forms, blood pressure machines, watches etc. Improper use leads to an increase in number of interventions that labouring women will go through, resulting in a more negative experience for them. Additionally, lack of adequate training on how to use the partograph also is a barrier to its use.

### Facilitators

Similarly, facilitators were found to contribute to all phases of delay particularly the first and second phases. Our finding that involvement of key community members—traditional leaders, religious leaders, and husbands— facilitate the use of preventive services concurs with a study which examined delays in accessing EmOC in Bangladesh; husbands were found to make half of the decisions on seeking care, as opposed to women deciding to seek care themselves in only one-eighth of the cases [33]. Furthermore, a study carried out in Zambia highlighted the importance of men being aware of and having a positive attitude towards the use of maternity waiting homes, as this results in them encouraging the use of such preventive services [34, 35]. Our findings also mirrored De Allegri’s study which identified the influence that village leaders carry in Burkina Faso. Through village leaders advocating for user fees reduction, a reduction occurred in the costs of facility deliveries [29]. Their involvement is a key facilitator as communities in which fistulas occur, are male centric societies in which they hold decision power.

Our findings showed that financial support through government partnerships, communities, and international donors served as a major factor in all three phases of the delay model through making resources available to labouring women, their communities, and health facilities they visit. Our study was in line with De Allegri et al. who highlighted that as a result of UN recommendations to subsidise obstetric care, there has been an increase in facility deliveries and a decrease in births at home [29].

### Strengths and limitations

To our knowledge, this review is the first to examine and synthesise barriers and facilitators associated with preventive interventions in sub-Saharan Africa, within the region with the highest prevalence of this injury. Moreover, it is the first to do so with the theoretical underpinning of the *Three Phases Delay Model*. Publication bias was reduced through a thorough search for unpublished literature using grey literature databases and websites of relevant organisations. A limitation of this review is its restriction to interventions in sub-Saharan Africa only. However, as Africa has the highest regional concentration of obstetric fistulas worldwide, the majority of interventions targets that population. Inclusion of descriptive studies also served as a limitation, as we were not able to carry out a full quality assessment due to the lack of an appropriate tool. We used our personal discretion when deciding whether to include a descriptive study, and were informed by the inclusion and exclusion criteria.

### Implications for practice and policy

- Future policies and initiatives should focus on increasing awareness of preventive interventions through training programs, and should not only be addressed to women but even more so to men and the leaders of communities.
- Simultaneously, the capability of health facilities to provide EmOC should be strengthened through appropriate and regular training on management of complicated labours, as well as through the provision of basic and essential tools and amenities.
- Lastly, financial support is required both in relation to the provision and payment of services, but also to improve transport and infrastructure, allowing labouring women to arrive sooner at facilities offering preventive care.

## Conclusion

This systematic review found that although barriers and facilitators were identified according to the phase of delay, they were found to play a role in all three phases of the delay model. Main barriers were found to be clustered and interlinked, while facilitators were less related. As the literature currently reflects a focus on preventive interventions aimed at either the decision to seek care or receiving adequate care, our review indicates that there is a need for more research on preventive interventions that target issues with regard to the second phase of delay, reaching a facility. Furthermore, there is a need to assess these preventive interventions as it will generate evidence reflecting their efficacy.

## Additional file

Additional file 1: Search strategy. (DOCX 16 kb)

## Abbreviations

AMSTAR: A measurement tool to assess systematic reviews
CASP: Critical Appraisal Skills Programme
EmOC: Emergency obstetric care
IGA: Income generating activities
SMAGs: Safe Motherhood Action Groups
WHO: World Health Organization

## References

[1] Adler A, Ronsmans C, Calvery C, Filippi V (2013). Estimating the prevalence of obstetric fistula: a systematic review and meta-analysis. BMC Pregnancy Childbirth.

[2] Bangser M (2006). Obstetric fistula and stigma. Lancet.

[3] Banke-Thomas A, Kouraogo S, Siribie A, Taddese H, Mueller J (2013). Knowledge of obstetric fistula prevention amongst young women in urban and rural Burkina Faso: a cross-sectional study. PLoS One.

[4] WHO (2014). 10 facts on obstetric fistula.

[5] Wegner M, Ruminjo J, Sinclair E, Pesso L, Mehta M (2007). Improving community knowledge of obstetric fistula prevention and treatment. Int J Gynaecol Obstet.

[6] Capes T, Ascher-Walsh C, Abdoulaye I, Brodman M (2011). Obstetric fistula in low and middle income countries. Mt Sinai J Med.

[7] Ramphal S, Moodley J (2006). Vesicovaginal fistula: obstetric causes. Curr Opin Obstet Gynecol.

[8] UNFPA. http://www.unfpa.org (2016). Accessed 19 Apr 2016.

[9] Fistula Foundation. http://fistulafoundation.org (2014). Accessed 19 Apr 2016.

[10] Cron J (2003). Lessons from the developing world: obstructed labor and the vesicovaginal fistula. MedGenMed.

[11] Banke-Thomas A, Wilton-Waddell O, Kouraogo S, Mueller J (2014). Current evidence supporting obstetric fistula prevention strategies in sub Saharan Africa: a systematic review of the literature. Afr J Reprod Health.

[12] Thaddeus S, Maine D (1994). Too far to walk: maternal mortality in context. Soc Sci Med.

[13] Markos D, Bogale D (2015). Documentation status of the modified World Health Organization partograph in public health institutions of bale zone, Ethiopia. Reprod Health.

[14] Gerten K, Venkatesh S, Norman A, Shu’Aibu J, Richter H (2009). Pilot study utilizing a patient educational brochure at a vesicovaginal fistula hospital in Nigeria, Africa. Int Urogynecol J Pelvic Floor Dysfunct.

[15] Ojanuga D (1991). Preventing birth injury among women in Africa: case studies in northern Nigeria. Am J Orthop.

[16] Ojanuga D (1992). Education: the key to preventing vesicovaginal fistula in Nigeria. World Health Forum.

[17] Tahzib F (1989). An initiative on vesicovaginal fistula. Lancet.

[18] Fistula C. Beyond repair: involving communities in fistula prevention and social reintegration—experience from Kissidougou, Guinea. New York: Fistula Care/EngenderHealth; 2010. https://fistulacare.org/archive/files/1/1.1/guinea_brief_beyond_repair.pdf. Accessed 20 Apr 2016.

[19] Fistula C. Integrating fistula treatment and prevention: the launch of a maternity unit in Sierra Leone. New York: Fistula Care/EngenderHealth; 2011. https://www.engenderhealth.org/files/pubs/fistula-care-digital-archive/2/2.2/Integrating-Fistula-Treatment-sierra_Leone_tech_brief.pdf. Accessed 20 Apr 2016

[20] Seim A, Alassoum Z, Bronzan R, Mainassara A, Jacobsen J, Gali Y (2014). Pilot community-mobilization program reduces maternal and perinatal mortality and prevents obstetric fistula in Niger. Int J Gynaecol Obstet.

[21] Turan J, Johnson K, Polan M (2007). Experiences of women seeking medical care for obstetric fistula in Eritrea: implications for prevention, treatment, and social reintegration. Glob Public Health.

[22] Miller S, Lester F, Webster M, Cowan B (2005). Obstetric fistula: a preventable tragedy. J Midwifery Womens Health.

[23] Nathan L, Rochat C, Grigorescu B, Banks E (2009). Obstetric fistulae in West Africa: patient perspectives. Am J Obstet Gynecol.

[24] Fistula C. Force MHT. Revitalizing the Partograph: does the evidence support a global call to action. New York: EngenderHealth/Fistula Care; 2012. https://fistulacare.org/wp-fcp/wp-content/uploads/pdf/program-reports/EngenderHealth-Fistula-Care-Partograph-Meeting-Report-9-April-12.pdf. Accessed 20 Apr 2016

[25] Fistula C. Improving partograph use in Uganda through coaching and mentoring. New York: Fistula Care/EngenderHealth; 2013. https://fistulacare.org/archive/files/2/2.3/Uganda_Partograph_technical_brief.pdf. Accessed 20 Apr 2016

[26] Levin K, Kabagema J (2011). Use of the Partograph: effectiveness, training, modifications, and barriers- a litearature review.

[27] Ngoma J. Prevention of Vesicovaginal fistula: a literature review and experience from Zambia. 2011. http://www.theseus.fi/handle/10024/26462; https://www.theseus.fi/bitstream/handle/10024/26462/Josephine%20ngoma%20thesis.pdf?sequence=1&isAllowed=y. Accessed 2 May 2016.

[28] UNFPA (2015). Obstetric fistula needs assessment report: findings from nine African countries.

[29] De Allegri M, Tiendrebeogo J, Muller O, Ye M, Jahn A, Ridde V (2015). Understanding home delivery in a context of user fee reduction: a cross-sectional mixed methods study in rural Burkina. BMC Pregnancy Childbirth..

[30] Melberg A, Diallo A, Tylleskar T, Moland K (2016). ‘We saw she was in danger, but we couldn’t do anything’: missed opportunities and health worker disempowerment during birth care in rural Burkina Faso. BMC Pregnancy Childbirth.

[31] Wall L, Karshima J, Kirschner C, Arrowsmith S (2004). The obstetric vesicovaginal fistula: characteristics of 899 patients from Jos, Nigeria. Am J Obstet Gynecol.

[32] Lori J, Munro-Kramer M, Mdluli E, Musonda G, Boyd C (2016). Developing a community driven sustainable model of maternity waiting homes for rural Zambia. Midwifery.

[33] Nahar S, Banu M, Nasreen H (2011). Women-focused development intervention reduces delays in accessing emergency obstetric care in urban slums in Bangladesh: a cross-sectional study. BMC Pregnancy Childbirth..

[34] Sialubanje C, Massar K, Kirch E, van der Pijl M, Hamer D, Ruiter R (2016). Husbands’ experiences and perceptions regarding the use of maternity waiting homes in rural Zambia. Int J Gynaecol Obstet.

[35] van Lonkhuijzen L, Stekelenburg J, van Roosmalen J (2012). Maternity waiting facilities for improving maternal and neonatal outcome in low-resource countries. Cochrane Database Syst Rev.

