# Supplementary material for: Barriers and facilitators to preventive interventions for the development of obstetric fistulas among women in sub-Saharan Africa: a systematic review
Source: BMC Pregnancy Childbirth. 2018 May 10;18:155. doi: 10.1186/s12884-018-1787-0 (PMC5946543; doi:10.1186/s12884-018-1787-0)
Supplement: Supplementary file 1 — Search strategy. (DOCX 16 kb) [file 12884_2018_1787_MOESM1_ESM.docx]

**Additional File 1: Search strategies**

PUBMED

| # | Add to builder | Searches | Results |
| --- | --- | --- | --- |
| #4 | Add | Search (((((vesico?vaginal fistula) OR recto?vaginal fistula) OR obstetric fistula)) AND prevent*) AND sub?saharan africa | 84 |
| #3 | Add | Search sub?saharan africa | 165793 |
| #2 | Add | Search prevent* | 1996368 |
| #1 | Add | Search ((vesico?vaginal fistula) OR recto?vaginal fistula) OR obstetric fistula | 4527 |

MEDLINE (OVID)

| # | Searches | Results |
| --- | --- | --- |
| 1 | fistula/ or exp vaginal fistula/ | 20751 |
| 2 | vesico?vaginal fistul*.mp. [mp=title, abstract, original title, name of substance word, subject heading word, keyword heading word, protocol supplementary concept word, rare disease supplementary concept word, unique identifier] | 2517 |
| 3 | recto?vaginal fistul*.mp. [mp=title, abstract, original title, name of substance word, subject heading word, keyword heading word, protocol supplementary concept word, rare disease supplementary concept word, unique identifier] | 1775 |
| 4 | obstetric fistul*.mp. [mp=title, abstract, original title, name of substance word, subject heading word, keyword heading word, protocol supplementary concept word, rare disease supplementary concept word, unique identifier] | 296 |
| 5 | 1 or 2 or 3 or 4 | 21434 |
| 6 | sub-saharan africa.mp. [mp=title, abstract, original title, name of substance word, subject heading word, keyword heading word, protocol supplementary concept word, rare disease supplementary concept word, unique identifier] | 12841 |
| 7 | exp "Africa South of the Sahara"/ | 160817 |
| 8 | 6 or 7 | 165938 |
| 9 | exp Preventive Health Services/ | 482393 |
| 10 | prevent*.mp. [mp=title, abstract, original title, name of substance word, subject heading word, keyword heading word, protocol supplementary concept word, rare disease supplementary concept word, unique identifier] | 1125667 |
| 11 | 9 or 10 | 1504115 |
| 12 | 5 and 8 and 11 | 63 |

EMBASE

| # | Searches | Results |
| --- | --- | --- |
| 1 | fistula/ or exp vaginal fistula/ | 79327 |
| 2 | vesico?vaginal fistul*.mp. [mp=title, abstract, original title, name of substance word, subject heading word, keyword heading word, protocol supplementary concept word, rare disease supplementary concept word, unique identifier] | 1814 |
| 3 | recto?vaginal fistul*.mp. [mp=title, abstract, original title, name of substance word, subject heading word, keyword heading word, protocol supplementary concept word, rare disease supplementary concept word, unique identifier] | 2746 |
| 4 | obstetric fistul*.mp. [mp=title, abstract, original title, name of substance word, subject heading word, keyword heading word, protocol supplementary concept word, rare disease supplementary concept word, unique identifier] | 452 |
| 5 | 1 or 2 or 3 or 4 | 79736 |
| 6 | sub-saharan africa.mp. [mp=title, abstract, original title, name of substance word, subject heading word, keyword heading word, protocol supplementary concept word, rare disease supplementary concept word, unique identifier] | 15670 |
| 7 | exp "Africa South of the Sahara"/ | 178638 |
| 8 | 6 or 7 | 184281 |
| 9 | exp Preventive Health Services/ | 23472 |
| 10 | prevent*.mp. [mp=title, abstract, original title, name of substance word, subject heading word, keyword heading word, protocol supplementary concept word, rare disease supplementary concept word, unique identifier] | 1603310 |
| 11 | 9 or 10 | 1603310 |
| 12 | 5 and 8 and 11 | 275 |

PSYCINFO

| # | Searches | Results |
| --- | --- | --- |
| 1 | fistula/ or exp vaginal fistula/ | 0 |
| 2 | vesico?vaginal fistul*.mp. [mp=title, abstract, original title, name of substance word, subject heading word, keyword heading word, protocol supplementary concept word, rare disease supplementary concept word, unique identifier] | 7 |
| 3 | recto?vaginal fistul*.mp. [mp=title, abstract, original title, name of substance word, subject heading word, keyword heading word, protocol supplementary concept word, rare disease supplementary concept word, unique identifier] | 5 |
| 4 | obstetric fistul*.mp. [mp=title, abstract, original title, name of substance word, subject heading word, keyword heading word, protocol supplementary concept word, rare disease supplementary concept word, unique identifier] | 19 |
| 5 | 1 or 2 or 3 or 4 | 30 |
| 6 | sub-saharan africa.mp. [mp=title, abstract, original title, name of substance word, subject heading word, keyword heading word, protocol supplementary concept word, rare disease supplementary concept word, unique identifier] | 2382 |
| 7 | exp "Africa South of the Sahara"/ | 0 |
| 8 | 6 or 7 | 2382 |
| 9 | exp Preventive Health Services/ | 0 |
| 10 | prevent*.mp. [mp=title, abstract, original title, name of substance word, subject heading word, keyword heading word, protocol supplementary concept word, rare disease supplementary concept word, unique identifier] | 176071 |
| 11 | 9 or 10 | 176071 |
| 12 | 5 and 8 and 11 | 1 |

COCHRANE LIBRARY

| # | Searches | Results |
| --- | --- | --- |
| #1 | MeSH descriptor: [Africa South of the Sahara] explode all trees | 4712 |
| #2 | MeSH descriptor: [Vaginal Fistula] explode all trees | 20 |
| #3 | prevention | 126968 |
| #4 | #1 and #2 and #3 | 3 |

WEB OF SCIENCE

| Basic Search | |
| --- | --- |
| obstetric fistula | |
| AND | sub-saharan africa |
| AND | prevent* |
